# Optical, Electrical, and Morphologic Characterization of Chromium-Impurified CdS Thin Films Elaborated by the Chemical Bath Deposition

**DOI:** 10.1021/acsomega.3c05003

**Published:** 2023-10-23

**Authors:** R. R. Contreras-Rodriguez, T. Mendivil-Reynoso, Isaias Ochoa Landin, S. J. Castillo, R. Ochoa-Landín

**Affiliations:** † Departamento de Física, Universidad de Sonora, Apdo. Postal 1626, Hermosillo, 83000 Sonora, Mexico; ‡ Universidad del Istmo Cd. Universitaria S/N, Sta. Cruz, 4ta. Sección, Sto. Domingo Tehuantepec, 70760 Oaxaca, Mexico; § Departamento de Investigación en Física, Universidad de Sonora, Apdo. Postal 5-088, Hermosillo, 83000 Sonora, Mexico

## Abstract

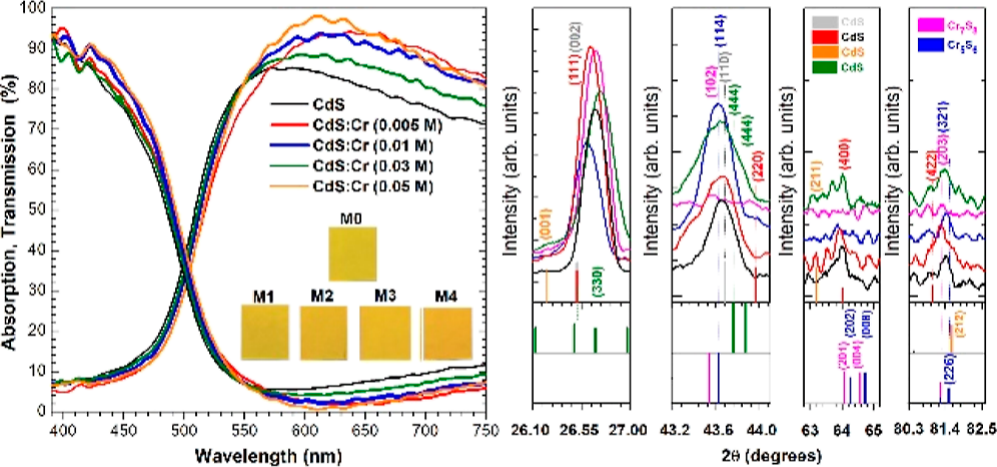

In this research work, a material system formed of cadmium sulfide combined with chromium atoms was developed to evaluate the influence of chromium concentration on the optical, electrical, structural, and morphological properties of a precursor layer of CdS. It is possible to observe that the transmission spectra increased for all chromium concentrations analyzed. From X-ray diffractograms, we conclude more accurately that CdS presents a mixture of phases, including orthorhombic, hexagonal, and cubic. Furthermore, the impact of adding chromium results in variations in the intensity of two major peaks in the diffractograms and an anomalous shift in the CdS pattern. The calculated resistivities show an invariable behavior of 4.5 × 10^6^ Ω cm. In addition, the bandgap values remain practically constant, with values of approximately 2.43–2.44 eV. The addition of chromium at different concentrations leads to surface morphology changes, as observed in SEM images.

## 1 Introduction

CdS thin films exhibit a high transparency in the visible spectrum and a band gap value of 2.45 eV. These make them a promising entrant for a wide range of applications, such as solar cells, optoelectronic devices, and sensors.^1−6^ Improving and controlling the properties of CdS films is compulsory if we want to make those devices more efficient.^7−10^ The results achieved during this investigation contribute to a deeper understanding of the effects of chromium doping on CdS thin films, stimulating the development of new devices and applications based on these materials.

There are several factors to be considered in order to reach the improvements previously mentioned. One way to guarantee it is through the proper growth of the film itself, which implies a resulting material with different characteristics, such as strains, morphology, porosity, and adherence, just to mention a few of them.

There are several techniques for growing thin films. Some of them are chemical in nature; sol–gel, chemical spray pyrolysis, chemical metal–organic vapor deposition, and chemical bath deposition. Others are physical in nature; electrodeposition, vacuum evaporation, sputtering, thermal evaporation, and spin coating.^11−13^ In addition, temperature, pressure, and pH are important parameters to consider in controlling the growth and morphology of the film. The chemical bath deposition technique (CBD) has various advantages. It is inexpensive and easy to handle, and it is possible to have a large deposition area of highly homogeneous film. In the CBD method, it is very important to control the pH because the film growth depends highly on this parameter.^14^

The purpose of this investigation was to explore doping techniques that are crucial for semiconductors to be used in practical devices. Holes in the valence band due to doping and specific treatments affect the conductivity of semiconductors. The presence of chromium as a doping agent in CdS thin films is expected to have positive effects on their properties.^15−17^ Investigations into CdS films doped with chromium (Cr) have been relatively scarce, as indicated in refs (18)–^20^.

In this work, the optical, electrical, structural, and morphological properties of CdS/Cr thin films are studied. Thus, with the aim of improving them and using them as a window layer in thin film solar cells of the heterojunction type, CdS:Cr/CdTe, a systematic study evaluating the influence of chromium concentration was carried out. The changes presented are observed in transmission spectra, X-ray diffraction patterns, and surface morphology. Electrical measurements were followed with a locally developed device. An UV–vis spectrophotometer, an X-ray diffractometer, and a Desktop SEM were used for the proper characterizations of the films.

## 2 Experimental Section

The chemical formulation developed in this research is presented and uses common and easy-to-handle chemical precursors. The films were deposited on substrates in a single deposition process by means of a low-temperature chemical bath deposition, which easily provides films with good adherence and uniform morphology. The reagent addition sequence is as follows: 10 mL of CdCl_2_ (0.05 M) is added into a beaker, followed by the same volume of K_2_CrO_4_ for different tested concentrations. Then, 20 mL of Na_3_C_6_H_5_O_7_ (0.5 M), 5 mL of KOH (0.3 M), and 10 mL of CS(NH_2_)_2_ (0.5 M) are added sequentially, and finally the sufficient quantity of deionized water to reach a total volume of 80 mL.

The reaction time and temperature were 3 h and 40 °C, respectively. For future reference, the samples made as explained above were labeled as follows: M0 = CdS, M1 = CdS:Cr (0.005 M), M2 = CdS:Cr (0.01 M), M3 = CdS:Cr (0.03 M), and M4 = CdS:Cr (0.05 M).

The deposited CdS films were characterized by using a UV–vis Ocean Optics 4000 spectrophotometer, an X-ray diffractometer (D2 PHASER BRUKER), Phenom ProX Desktop SEM were used, and the electrical measurements were implemented with a device developed at the University of Sonora, whose capability has been successfully tested and published.^17^

## 3 Results and Discussion

**Figure 1:**
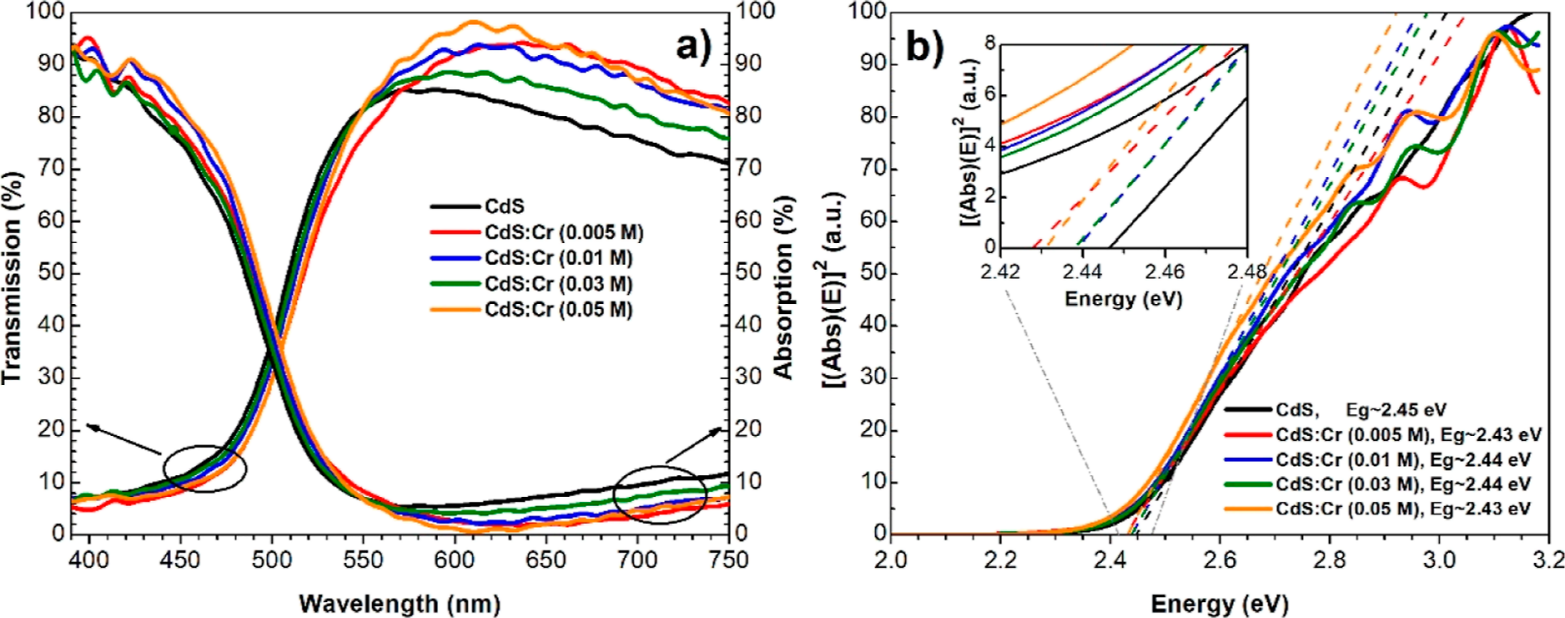
(a) Presents both the transmission and absorption spectra for all CdS films doped with chromium, in comparison with that of the undoped CdS film. (b) Corresponding to band gaps evaluation for the complete serie of samples of CdS and CdS:Cr at four different concentrations.

On one hand, Figure 1a depicts cadmium sulfide (CdS) thin film transmission spectra before and after doping with chromium at different concentrations for comparative purposes. It can be observed how the transmission spectra of all CdS:Cr films exceed that of pure CdS film for wavelengths greater than 570 nm. It is possible that the doping effect caused a directional shift in the electromagnetic radiation toward the transmission half-plane. The absorption spectra show a complementary trend.

Figure 1b, on the other hand, illustrates the graphical behavior of band gaps for the complete series of samples of CdS and CdS:Cr at the four different concentrations mentioned above. The direct band gap values were estimated using the Tauc method. The pure CdS sample exhibited a band gap value of *E*_g_ = 2.45 eV. This value coincides with that reported in the scientific literature.^18−23^

For samples M1 and M4, a slight decrease of two hundredths of 1 eV was observed. Samples M2 and M3 showed a decrease of one hundredth of 1 eV, which was to be expected due to the doping introduced by the carriers, causing the band gap to be reduced. The fluctuations present at the band gap plot for higher energies suggest intraband gap states. These states could be due to doping, a lattice defect, bulk behavior, or surface modification.

**Figure 2:**
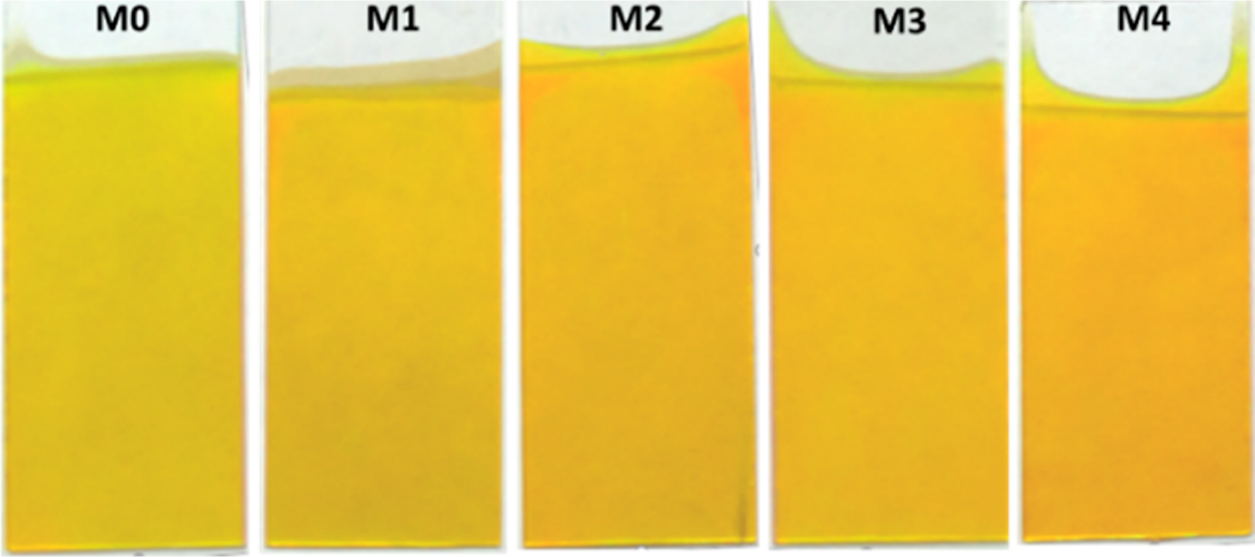
M0 corresponds to the coloration of the reference material. The concentration of chromium increases in the samples, and its hue intensifies toward an orange color.

Figure 2 shows a sequence of images of all of the synthesized samples. Starting with the film of pure CdS (M0) on the far left and continuing with the films doped at different chromium concentrations: 0.005, 0.01, 0.03, and 0.05 M in ascending order from left to right, respectively. It can be observed the different color tonalities.

**Figure 3:**
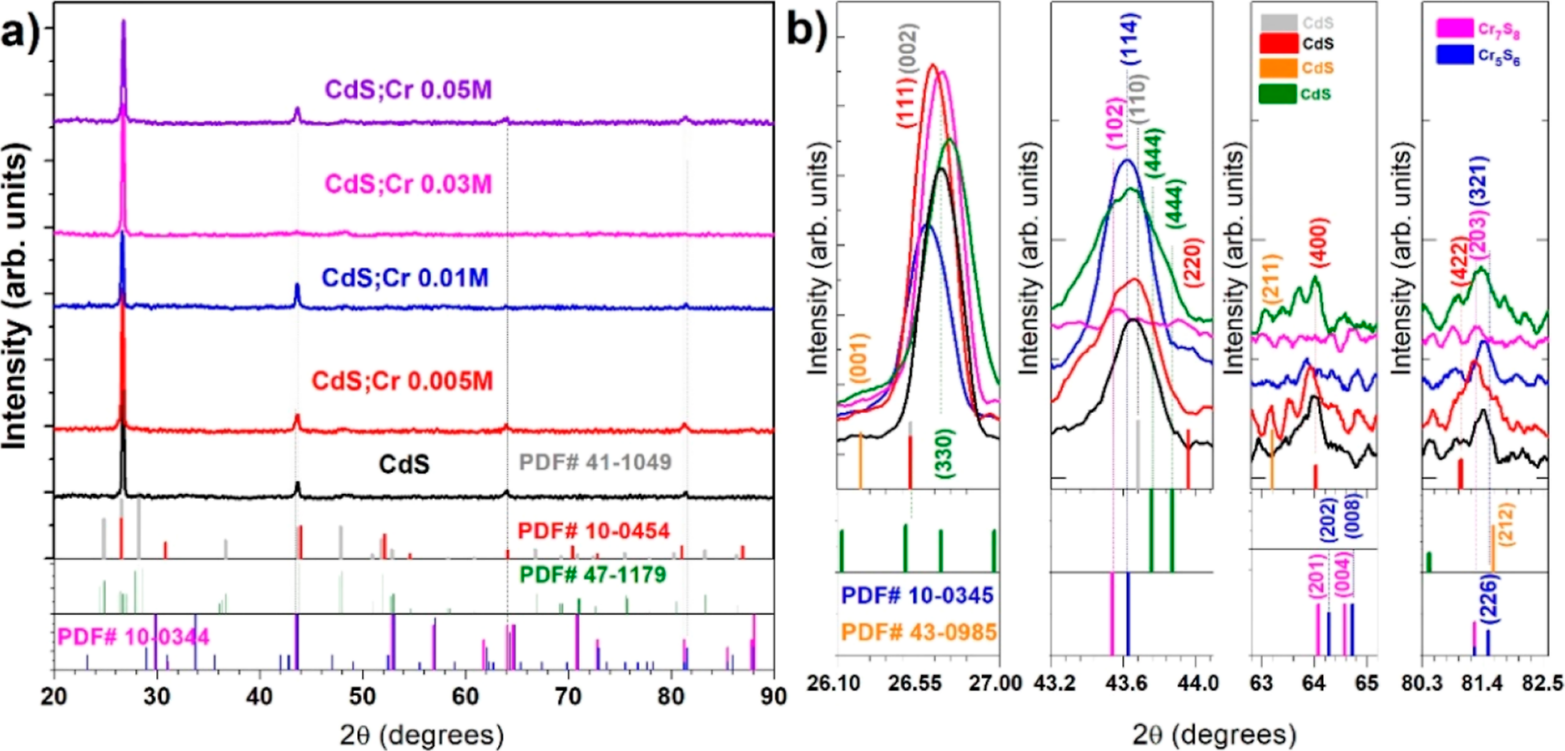
In (a), the graphs corresponding to the diffraction patterns of all CdS:Cr and CdS films are presented, while in (b), translations and magnifications were performed in order to determine the precise shift of the maximum peak intensities.

The X-ray diffraction characterization is presented in Figure 3. In part a, the experimentally obtained diffraction patterns are shown from 2θ = 20 to 90°. The arrangement of the diffraction patterns is presented in ascending order. Starting with the pure CdS thin film (M0), followed by the sample (M1) CdS:Cr (0.005 M), then (M2) CdS:Cr (0.01 M), (M3) CdS:Cr (0.03 M), and finally (M4) CdS:Cr (0.05 M).

Matching experimental patterns with a database of powder diffraction patterns (PDF). These lines have been placed at the lower parts of the patterns in Figure 3a,b. In the upper right part of Figure 3b, colored labels are placed to associate them with the identified compounds and their PDF codes. PDF# 47-1179 CdS, PDF# 10-0454 CdS, PDF# 41-1049 CdS, PDFF# 43-0985 CdS, PDF# 10-0344 Cr_7_S_8_, and PDF# 10-0345 Cr_5_S_6_. It can be observed that these patterns exhibit four peaks, with their most intense peaks at approximately 26.55 and 43.6. The two diffraction peaks at lower angular values allow one to perceive changes in their relative intensities. There is no correlated trend between the intensity changes of these two peaks and the concentrations of chromium used in the synthesis.

Based on a preliminary assessment using Figure 3a, the usual approach would be to identify the CdS starting material as having a cubic structure. Any peak shift is attributed to stretching or compression of the lattice. However, in Figure 3b, partial magnifications of each of these four peaks are presented within an angular range where they are centered. Here, the corresponding shifts between the different synthesized samples can be appreciated. Under scrutiny, the reference material, chromium-free CdS, with reported patterns, was identified. It can be seen that the first peak is shifted to the right of the cubic value and the second peak is shifted to the left, while the first peak coincides with the Miller index (330) of an orthorhombic phase and the second peak coincides with the Miller index (110) of a hexagonal phase. Upon analysis of the next two peaks, it was observed that the third one precisely coincides with the Miller index (400) for cubic CdS. Finally, the last peak analyzed for the pure CdS sample lies between two phases, closer to a cubic one (422), and further from another orthorhombic phase (212). The above observations, along with the experimental X-ray diffraction patterns, suggest that four different mixtures of CdS crystals coexist in the samples. However, the presence of chromium in the first peak of the subsequent samples could not be evaluated because the characteristic signals of fundamental compounds with chromium are outside the chosen interval for the main peak. Nevertheless, in the second peak shown between 43.2 and 44°, the shifts are very close, surrounding the value 43.6°. This can be associated with the presence of chromium sulfide formations Cr_7_S_8_ and Cr_5_S_6_.

**Figure 4:**
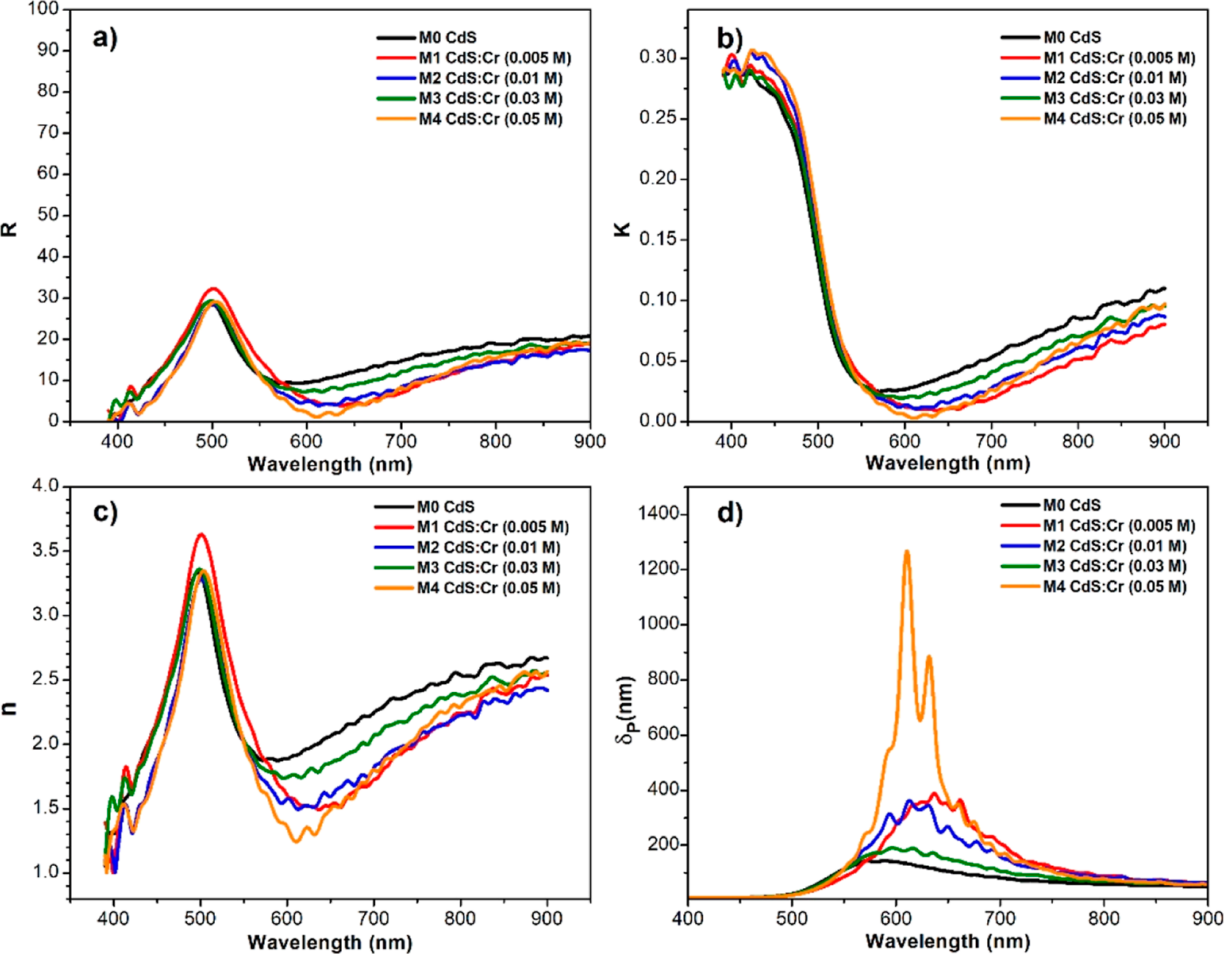
(a) Shows reflection spectra of CdS:Cr and CdS samples versus wavelength. (b) Displays extinction coefficient variation for the same samples versus wavelength. (c) Depicts refractive indices of all samples, showing different rates of change linked to light speed variations. Finally, (d) presents the depth of visible light penetration for synthesized CdS and CdS:Cr samples at (1/*e*) attenuation.

Optical properties such as reflection, extinction coefficient, refractive index and deep penetration of light were estimated. Similar studies for CdS have been published in refs (24−28). Reflection is determined through a balance or conservation equation, as stated in eq 1. In Figure 4a, it is observed that the highest value for all reflection curves is approximately 33% for wavelengths close to 500 nm. While the behavior decreases for wavelengths lower or higher than 500 nm. All reflection curves gradually increase, starting from around 620 nm up to 20%.^26,27,30−33^

The extinction coefficient of light shows the medium’s ability to absorb or scatter light in a correlated manner. A high extinction coefficient results in increased light scattering within the medium. Whereas a low extinction coefficient leads to greater light absorption. Figure 4b displays the extinction coefficients of all CdS and CdS:Cr samples. Their behavior decreases within the range of 400–550 nm, with a maximum value of approximately 0.30. From 550 to 900 nm, the extinction coefficients exhibit an upward trend up to a value of 0.10.

Figure 4c presents the refractive indices of all CdS and CdS:Cr films. Clearly, the refractive index values range between 1.0 and 3.75 for wavelengths ranging from 400 to 500 nm. However, beyond 500 nm, the indices decreased once again.

To find the values of the extinction coefficient (*k*) and of the refractive indices (*n*), considering the transparency of the films, eqs 2 and 3 were used.

The subsequent analysis explores the depth of visible light penetration as a function of wavelength. For this purpose, was used a developed mathematical model found in the scientific literature, corresponding to eq 4. Figure 4d depicts the graphs for our developed materials CdS and CdS:Cr. As shown by all the samples, they exhibit a band between 480 and 750 nm. All samples doped with Cr exhibit greater light penetration compared to pure CdS. Sample M4, corresponding to the highest chromium concentration, shows a maximum penetration value at a wavelength of 614 nm.
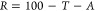
1
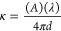
2
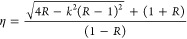
3
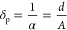
4

**Figure 5:**
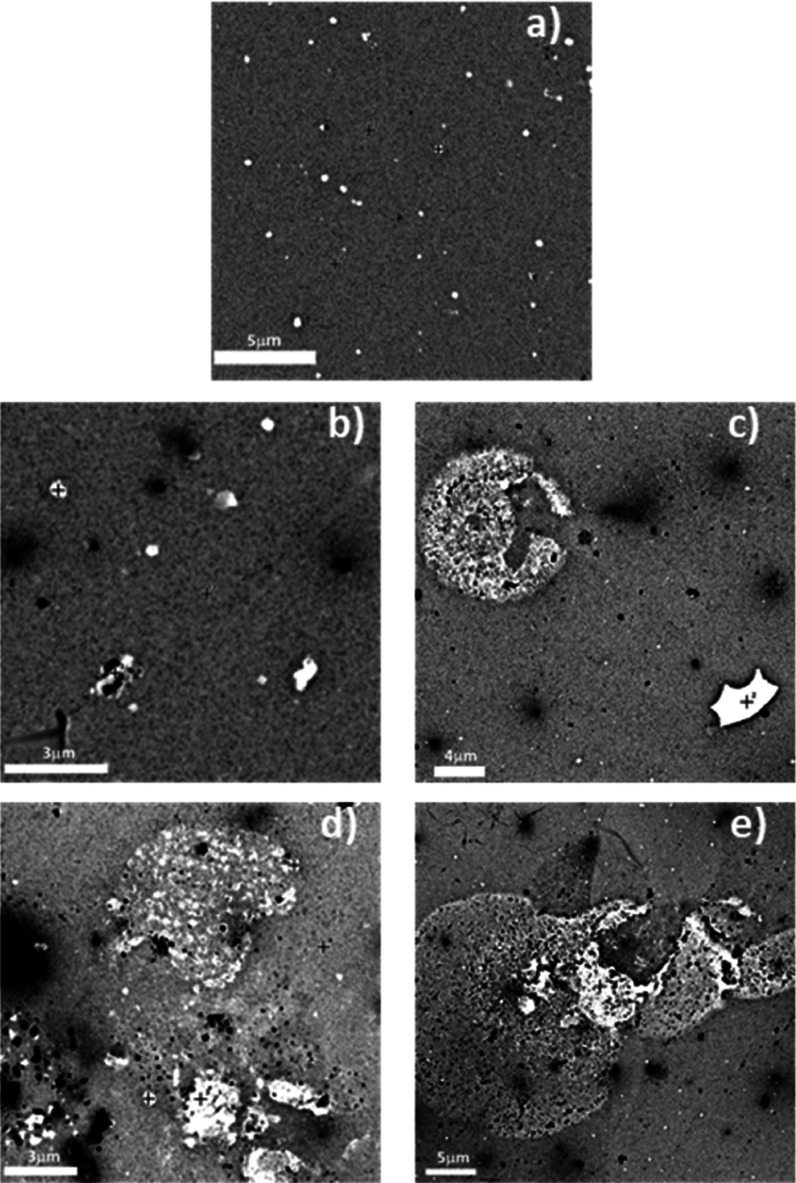
SEM images are used to describe the surface morphology. CdS (a) and CdS:Cr thin films for different concentrations, 0.005, 0.01, 0.03, and 0.05 M, which correspond to (b–e), respectively.

In Figure 5, the surface morphology of the complete set of thin films is depicted. Part (a) corresponds to the thin film of pure CdS or precursor material, where a flat background is observed along with round CdS clusters. Micrograph (b) exhibits magnification as well as formations of clusters and the distribution of pinholes. Images (c–e) illustrate the formation of large islands on a flat background.

The increase in the chromium concentration is believed to be closely associated with the observed changes in the surface morphology of the analyzed samples. To substantiate this argument, observe the percentage proportions of the chromium element in Table 1 achieved by EDS.

**Table 1:** Compositional Matrix of the Synthesized Films and the Glass Substrate

element	atomic (%) M0	atomic (%) M1	atomic (%) M2	atomic (%) M3	atomic (%) M4
Si	34.3	27.9	39.1	33.3	32.5
Cd	31.3	31.5	21.8	29.7	27.6
Ca	5.6	3.8	6.6	5.2	5.0
S	7.2	7.0	5.0	6.9	6.3
Na	4.2	4.7	4.8	4.3	4.8
O	13.0	21.4	18.8	15.8	18.8
Mg	1.9	2.1	2.3	1.9	2.1
Br	2.9	1.3	1.3	2.4	2.5
Cr	0.0	0.3	0.3	0.4	0.4

We consider that a predominant reaction mechanism is followed in the growth process of CdS films, even when excess proportions of chromium are added in the interval of our concentrations.

Table 1 shows additionally chemical elements in the Corning glass substrate used to grow the CdS and CdS:Cr films. Therefore, the additional elements of Cd, S, and Cr are due to this substrate.

**Figure 6:**
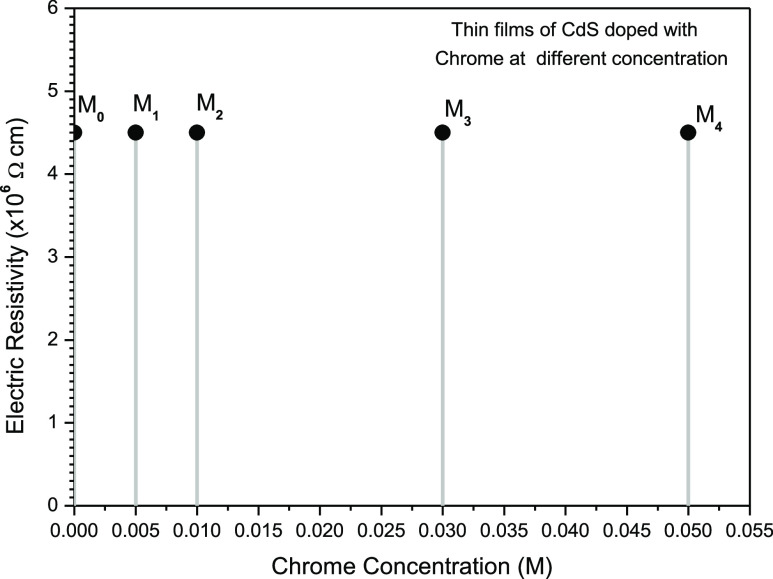
Proof of constant behavior of electrical resistivity for complete set of samples M0 until M4.

Electrical resistances were realized to calculate the electrical resistivity of all samples. Figure 6 reveals the constant behavior of all samples, regardless of chromium concentrations.

## 4 Conclusions

All of the synthesized films are homogeneous and have good adhesion to the substrate.

For wavelengths greater than 570 nm, the transmission spectra of all CdS:Cr films surpass those of the pure CdS film. The band gap values remain practically constant, with values of approximately 2.43–2.44 eV.

From the experimental X-ray diffraction patterns, four different phases of CdS crystals. There is evidence of the existence of Cr_7_S_8_ and Cr_5_S_6_ chromium sulfide formations.

The results show that all the reflection curves present a band between 400 and 620 nm, with a maximum of 500 nm. All reflection curves gradually increase from about 620 nm up to 20% reflection.

Light penetration depth curves of all samples prove that CdS:Cr (0.05 M) exhibits the highest penetration of 570–750 nm.

The SEM images show significant changes in the surface as the chromium dopant concentration increases. The formation of clustered agglomerates in the shape of islands is promoted. We also associate this phenomenon with the formation of chromium sulfides.

The calculated resistivities show an invariable behavior of 4.5 × 10^6^ Ω cm.

A different interpretation for peak shifts in X-ray diffraction patterns in thin film synthesis by CBD is given. Those are commonly attributed to compression and stretching of the lattice. The predominant mechanism in a chemical reaction may present uncertainties. It leads to the formation of products of different stoichiometries. Therefore, mixtures of crystals can be achieved in a lower proportion, along with their consequences.^1,29^
